# Understanding the Boundaries of Empathy: A Qualitative Exploration in Pediatric Oncology Nursing †

**DOI:** 10.3390/children13080984

**Published:** 2026-07-24

**Authors:** Ayfer Aydın, Merve Ertunç Soycan, Özlem Şensoy, Hülya Dalkılıç Bingöl, Rejin Kebudi

**Affiliations:** 1 Department of Nursing, Faculty of Health Sciences, Istanbul Atlas University, Istanbul 34408, Türkiye; 2 Department of Pediatric Nursing, School of Nursing, Istanbul University, Istanbul 34116, Türkiye; merve.ertunc@istanbul.edu.tr (M.E.S.); ozlem.sensoy@istanbul.edu.tr (Ö.Ş.); 3 Department of Pediatric Hematology-Oncology, Institute of Oncology, Istanbul University, Istanbul 34093, Türkiye; h.bingol@istanbul.edu.tr; 4 Department of Pediatric Hematology-Oncology, VKV Amerikan Hospital, School of Medicine, Koç University, Istanbul 34365, Türkiye; rkebudi@ku.edu.tr

**Keywords:** empathy, nursing, pediatric oncology, qualitative research

## Abstract

**Highlights:**

**What are the main findings?**

- Pediatric oncology nurses consider empathy an essential component of high-quality nursing care and describe it as a skill that develops through clinical experience.
- Nurses experience challenges in balancing empathic engagement with professional boundaries, while excessive emotional involvement may contribute to compassion fatigue and emotional exhaustion.

**What are the implications of the main findings?**

- Findings highlight the need for structured empathy training programs that support nurses in developing empathic skills while maintaining professional boundaries.
- Strengthening organizational and educational support for empathy may help improve nurse well-being, reduce emotional burden, and enhance the quality of pediatric oncology care.

**Abstract:**

Background/Objectives: Empathy is a fundamental component of pediatric oncology nursing. This study aims to examine in depth how nurses working in pediatric oncology define empathy, experience it in clinical practice, and manage emotional and professional boundaries in the empathetic care process. Methods: A qualitative research design was used. Semi-structured individual interviews were conducted with 17 nurses with at least 2 years of experience in pediatric hematology-oncology units. The interviews were conducted via Zoom. Data were analyzed using Braun and Clarke’s thematic analysis, and data collection continued until thematic saturation was achieved. Results: Three main themes were identified: Understanding Empathy, The Journey of Empathy, and The Cost of Empathy. Nurses defined empathy as an indispensable part of high-quality pediatric oncology care; however, they reported conceptual uncertainty in distinguishing empathy from sympathy and indecision regarding appropriate emotional boundaries. Empathy was perceived as a dynamic process shaped more by clinical experience than formal education. Early professional experiences were often characterized by emotional distress, while increasing experience enabled nurses to regulate their emotions and adopt more sustainable empathic practices. Despite this adaptation, prolonged exposure to children’s suffering and death was associated with compassion fatigue and emotional exhaustion. The absence of structured empathy training and formal emotional support systems further intensified these challenges. Conclusions: In pediatric oncology nursing, empathy is a complex, evolving professional competency that demands continuous emotional regulation and boundary management. Structured empathy training, reflective supervision, and strengthening institutional support mechanisms are essential to promote sustainable empathic care while protecting nurses’ emotional well-being.

## 1. Introduction

Pediatric cancer diagnosis and treatment create a care environment marked by uncertainty, fear, anxiety, grief, and prolonged emotional strain for children and families [1,2,3]. In this environment, pediatric oncology nurses must respond simultaneously to complex clinical needs and recurrent emotional distress across the treatment trajectory. Because they provide continuous bedside care, nurses are often central to recognizing emotional cues, facilitating communication, and sustaining trust during treatment, deterioration, and bereavement. Open communication and compassionate responses are therefore integral not only to the experiences of children and families but also to the quality of pediatric oncology nursing care [4,5].

Among healthcare professionals, nurses hold a unique position, providing continuous, holistic care that integrates clinical competence with emotional support. Within this role, empathy can be understood as the capacity to recognize and understand another person’s perspective and emotional state, communicate that understanding, and respond in a clinically appropriate manner while retaining professional judgment [4,6,7]. Studies have shown that empathic communication enhances treatment adherence, strengthens nurse–family relationships, and improves overall satisfaction and psychosocial outcomes [6,8,9].

Empathy in nursing is multidimensional and commonly categorized into cognitive and emotional dimensions [10,11]. Cognitive empathy refers to intellectually understanding another person’s emotional state, while emotional empathy involves sharing those feelings on an affective level [6,12]. Effective clinical empathy, however, also requires translating this understanding into a helpful response and regulating one’s own emotional involvement. In healthcare, empathy is frequently conflated with sympathy and compassion, though these concepts are distinct [13,14]. Sympathy may involve sharing another person’s distress and can increase the risk of over-identification, whereas compassion includes concern and a motivation to alleviate suffering [15,16]. Empathy requires closeness sufficient for understanding without losing the reflective distance needed for sound clinical judgment [4]. Professional boundaries should therefore be viewed not as the opposite of empathy, but as one of the conditions that can make empathic care therapeutic and sustainable.

The development of empathy is therefore better understood as a “journey of empathy” than as the simple accumulation of years in practice. Clinical exposure can help nurses recognize emotional cues, anticipate family needs, regulate their own responses, and establish more workable boundaries; however, repeated exposure does not automatically produce deeper or more skillful empathy [9,17]. Without opportunities for reflection and support, experience may also foster emotional suppression, defensive distancing, or normalization of suffering, while prolonged exposure to deterioration and death may contribute to compassion fatigue and emotional exhaustion [2,18,19]. Thus, an apparently calmer response among more experienced nurses may represent adaptive emotional regulation, but it may also reflect self-protective detachment; these possibilities require critical examination rather than assuming that empathy increases linearly with seniority.

The journey of empathy is likely shaped by interactions among personal dispositions, age, and broader life experience, education, reflective capacity, mentoring, clinical encounters, team culture, workload, and access to organizational support. Structured education, simulation, reflective practice, and communication training can strengthen perspective-taking and empathic responding, while supervision, peer support, and staff well-being initiatives can help nurses process emotionally demanding encounters [20,21,22,23,24]. Accordingly, chronological age and professional tenure should not be treated as interchangeable or sufficient indicators of empathic competence; nurses at similar career stages may follow different developmental paths depending on the learning and support available to them [9,25].

Although empathy is widely recognized as fundamental to high-quality care, limited research has critically examined how pediatric oncology nurses understand changes in their empathy over time, distinguish professional growth from emotional self-protection, and attribute this development to clinical experience versus personal, educational, and organizational influences [12,26]. Understanding the journey of empathy from nurses’ perspectives is essential for developing interventions that foster reflective, bounded, and sustainable empathic engagement while safeguarding emotional well-being. Therefore, this qualitative study aimed to explore how pediatric oncology nurses understand and practice empathy, how they describe its development across their professional journey, and how they negotiate emotional and professional boundaries within the demanding clinical context of pediatric oncology care.

## 2. Methods

### 2.1. Study Aim

This descriptive phenomenological qualitative study aims to explore and provide an in-depth understanding of pediatric oncology nurses’ experiences of empathy.

### 2.2. Participants

The sample consisted of 17 nurses. The inclusion criteria were as follows: Nurses had to have at least two years of experience working in pediatric hematology-oncology units, have provided care for children with cancer, and be willing to volunteer for the study. Participants were employed in pediatric hematology-oncology units at public, university, and private hospitals in Türkiye; therefore, the sample comprised nurses from different institutional settings rather than a single hospital.

### 2.3. Data Collection

Participants were recruited through purposive sampling. Potential participants who met the inclusion criteria were identified across pediatric hematology-oncology settings in public, university, and private hospitals in Türkiye and invited to participate voluntarily. Eligibility was confirmed before an interview was scheduled. Data were collected through individual semi-structured interviews conducted via Zoom by the first author (AA). With participants’ consent, all interviews were digitally recorded.

The semi-structured interview guide contained seven main open-ended questions: (1) “What does empathy mean to you in the context of pediatric oncology nursing?” (2) “Can you describe a situation in which you felt that you were being empathetic toward a child with cancer or the child’s parents?” (3) “How do you distinguish empathy from sympathy and from professional behavior in your practice?” (4) “Has your understanding or practice of empathy changed over the course of your professional experience? Please describe.” (5) “What experiences or factors have influenced the way you use empathy in care?” (6) “How does empathic engagement affect you emotionally and professionally?” and (7) “What do you do to protect your well-being and maintain professional boundaries while remaining empathetic?” Clarifying and exploratory probes, such as “Can you explain this further?” and “Can you provide an example?”, were used when necessary.

AA is a nurse academic and professor with master’s and doctoral training in Child Health and Disease Nursing and extensive experience in pediatric nursing, pediatric oncology research, and qualitative interviewing. Her relationship with participants was limited to recruitment and interview scheduling; she had no clinical, managerial, or supervisory role in relation to them. Her professional familiarity with pediatric oncology was acknowledged as a potential influence on data generation and interpretation. To support reflexivity, interpretations were compared and discussed collaboratively within the research team throughout coding and theme development.

There are varying perspectives in the literature regarding the appropriate sample size for qualitative research. Therefore, interviews were continued until no new codes or information emerged, indicating data saturation. After conducting 17 interviews with nurses, no new data or themes were identified, and data saturation had been achieved.

### 2.4. Data Analysis

Thematic analysis was conducted following the framework of Braun and Clarke [27]. The process involves:

1. Familiarization: Interviews were transcribed (MES), then two researchers (AA, MES) repeatedly read the transcripts to immerse themselves in the data.
2. Generating Initial Codes: The researchers identified initial codes that captured key aspects of nurses’ experiences of empathy and associated challenges.
3. Identifying Themes: Codes were grouped and organized into preliminary themes.
4. Reviewing Themes: Themes were refined collaboratively to resolve differences and highlight similarities.
5. Defining and Naming Themes: Three researchers (HDB, MES, ÖŞ) finalized the themes and subthemes.
6. Producing the Final Report: The final themes and subthemes were reviewed by all researchers for feedback and suggestions.

### 2.5. Trustworthiness

The trustworthiness of the study was established according to Lincoln and Guba’s (1985) criteria, including credibility, dependability, confirmability, and transferability [28]. To ensure credibility, three researchers analyzed the data independently and collaboratively through repeated readings of the transcripts to deepen their understanding. Peer debriefing and investigator triangulation were also used to enhance the accuracy of interpretations. Dependability was maintained by conducting all interviews with the same experienced researcher (AA), using a consistent semi-structured interview guide, and ensuring a stable data-collection process.

Confirmability was achieved through systematic documentation of analytical decisions, maintaining an audit trail, and supporting interpretations with representative quotations from participants.

To promote transferability, the study provided a detailed description of the research context, participant characteristics (nurses), and variations in perspectives, allowing readers to assess the applicability of the findings to other contexts. Finally, the study followed the COREQ checklist to ensure transparency and rigor in reporting qualitative research procedures [29].

### 2.6. Ethical Considerations

This study was performed in line with the principles of the Declaration of Helsinki. Ethical approval was obtained from the University Social and Humanities Research Ethics Committee (No: 2025/132). Interviews were conducted after receiving verbal and written consent. Participants were informed that they had the right to withdraw their consent at any time without any justification.

## 3. Results

The study included 17 female participants, with a mean age of 28.89 years (SD = 3.91, range: 24–36). The interviews lasted approximately 35–45 min. Most participants held a bachelor’s degree (88.2%), and work experience in pediatric oncology units ranged from 2 to 5 years (41.2%) to more than 10 years (23.4%). The majority were employed in private hospitals (64.3%). Participant characteristics are presented in Table 1.

**Figure 1:**
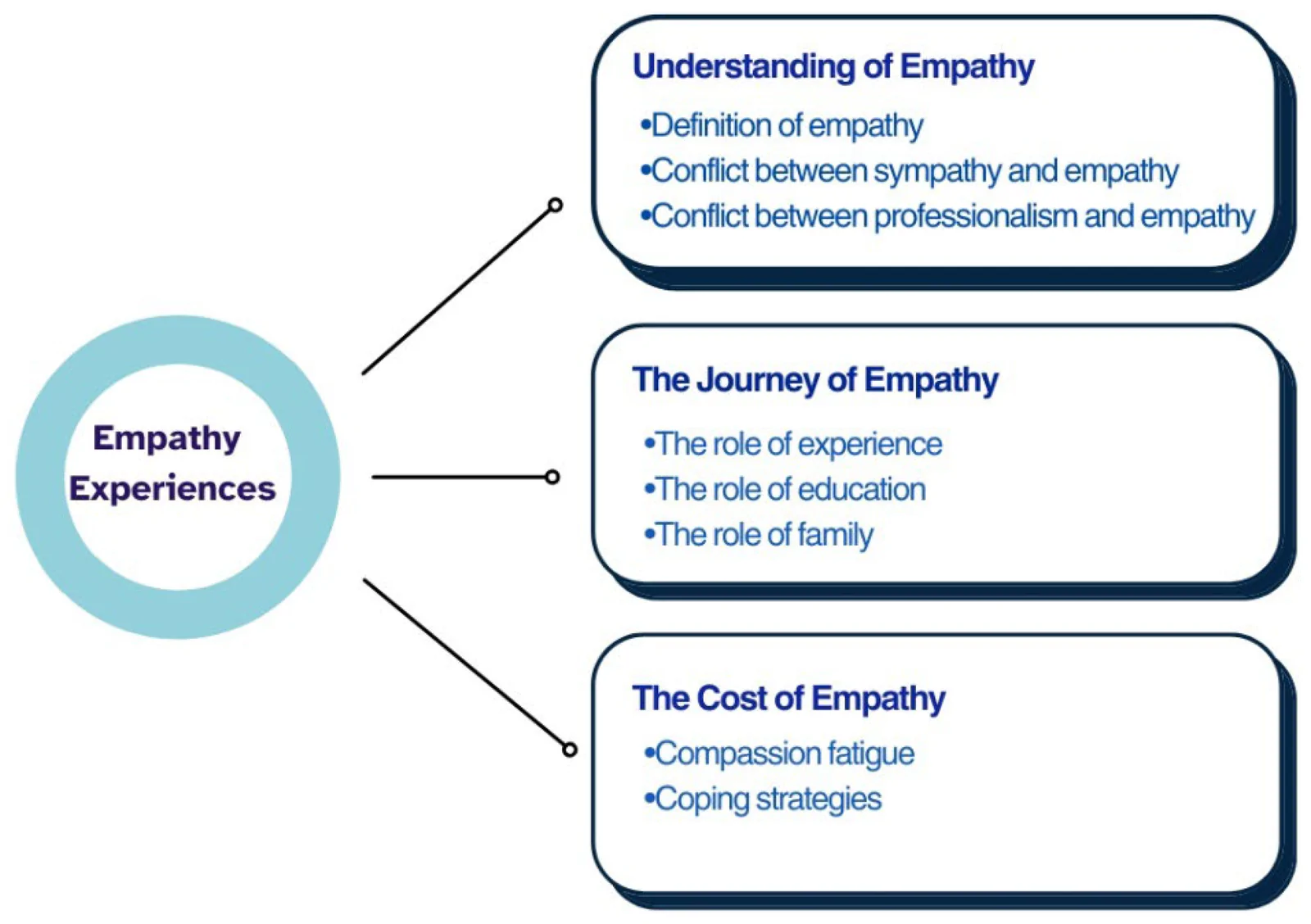
Themes and subthemes.

Three main themes were identified: Understanding Empathy, the Journey of Empathy, and the Cost of Empathy. Three main themes and subthemes emerged, shown in Figure 1.

### 3.1. Understanding of Empathy

In this study, nearly all nurses emphasized the importance of empathy in pediatric oncology, noting that it is an essential quality for nurses. However, notable ambiguity emerged between the concepts of empathy and sympathy, as well as between empathy and professional behavior. Many nurses expressed uncertainty about where empathy begins, when it transitions into sympathy, and whether excessive empathy might be perceived as unprofessional.

#### 3.1.1. Definition of Empathy

When asked about the meaning of empathy, most nurses provided textbook definitions, with nearly all describing it as “putting oneself in the patient’s shoes.” While some nurses took an emotional approach to defining empathy, others adopted a more objective stance. Most define empathy as “the ability to emotionally understand what other people are feeling, to see things from their perspective, and to place oneself in their position.”

*“It’s about understanding how the person in front of you might react in a specific situation. Let’s say a patient is in pain—empathy means believing their pain is real, even if you’re not experiencing it.”*

(Nurse 1, 2 years of experience)

*“Empathy is the ability to recognize what others feel and resonate with those emotions within ourselves.”*

(Nurse 8, 4 years of experience)

*“For me, empathy is placing yourself in someone else’s shoes and showing them that you understand their emotional state.”*

(Nurse 10, 7 years of experience)

*“I always tell nurses: ‘That person in that bed is not a stranger. Treat them as if they were your most beloved family member.’ That’s what empathy means to me—caring as though the patient were someone I deeply love.”*

(Nurse 15, 14 years of experience)

#### 3.1.2. Conflict Between Sympathy and Empathy

However, as the interviews progressed, it became evident that they experienced conceptual confusion about empathy and sometimes struggled to decide how to act in practice. Nurses frequently reported confusion between sympathy and empathy. Sympathy was described as more emotionally charged and potentially intrusive, while empathy was viewed as a more professional and therapeutic form of engagement. Nurses often expressed uncertainty about whether their approach to patients reflected empathy or sympathy, and about where the boundaries of empathy begin and when it transitions into sympathy.

*“I used to think I was being empathetic, but it was sympathy. Now my approach is more balanced and truly empathic.”*

(Nurse 5, 3 years of experience)

*“Sympathy can be valuable, but to effectively fulfill our professional responsibilities, we often need to set our emotions aside and approach situations in a more solution-focused, practical, and efficient manner… Once we become too immersed in those emotions, it can be challenging to maintain control. While it is important to acknowledge and understand emotions, their heightened intensity can also take a toll on our well-being.”*

(Nurse 10, 7 years of experience)

#### 3.1.3. Conflict Between Professionalism and Empathy

Nurses expressed the challenges they faced in balancing empathy with professional detachment. Although they viewed empathy as vital for providing meaningful care, they also voiced concerns about becoming overly emotionally involved. Some nurses stated that they consciously suppressed their emotional responses, such as holding back tears in front of families. Another nurse explained that, as a professional approach, she addressed all mothers as “mother” rather than by name.

*“Empathy is putting yourself in someone else’s place; sympathy involves emotional closeness that may disrupt professional boundaries.”*

(Nurse 12, 5 years of experience)

*“You don’t need to get too close to the patient’s family.”*

(Nurse 3, 2 years of experience)

*“Some patients may naturally be more likable, but becoming too close and crossing professional boundaries is not appropriate. …. As sympathy deepens, it can lead to greater emotional strain on the nurse. In contrast, empathy maintains a professional distance while still allowing for understanding and support.”*

(Nurse 15, 14 years of experience)

### 3.2. The Journey of Empathy

Most nurses reported that their empathy skills had evolved over the years, highlighting experience as the primary factor influencing this development. They also noted that both undergraduate education and in-service postgraduate training contributed to their awareness of empathy.

#### 3.2.1. The Role of Experience

Nurses emphasized that clinical experience plays the most pivotal role in shaping and strengthening empathy. Many nurses described their early professional years as emotionally overwhelming, characterized by confusion and an inability to respond effectively to the emotional needs of patients and families. Over time, through repeated encounters with suffering, grief, and complex care dynamics, they learned to manage their emotional responses, develop clinical insight, and establish a more stable and constructive empathic approach.

*“At first, I was very emotional… Now I approach things more rationally.”*

(Nurse 9, 7 years of experience)

*“In the beginning, I didn’t know what to do—I just froze. Now I respond more effectively.”*

(Nurse 2, 14 years of experience)

*“I used to stare blankly. Now I understand treatment processes and can empathize better with both patient and family.”*

(Nurse 13, 12 years of experience)

#### 3.2.2. The Role of Education

While nurses acknowledged the presence of some communication-related courses during undergraduate education, they consistently highlighted the absence of structured empathy training. Empathy was often touched upon superficially within broader topics such as patient-centered care or professional communication. Furthermore, in-service training focused on emotional competencies or empathic care was largely lacking.

*“We had some courses on communication and psychology in undergrad.”*

(Nurse 3, 2 years of experience)

*“There was no specific empathy training in my career.”*

(Nurse 16, 7 years of experience)

#### 3.2.3. The Role of Family

Family experiences emerged as a meaningful factor influencing nurses’ empathy development, particularly in relation to emotional awareness and regulation. Nurses recalled that, early in their careers, they frequently shared distressing patient-related experiences with family members. Family members’ responses helped some nurses recognize the intensity of their emotional burden and the extent to which work-related distress was spilling into home life.

Participants subsequently described sharing these stories less frequently. They associated this change with adapting to the emotional demands of pediatric oncology and developing clearer boundaries between work and home. However, their accounts did not establish whether reduced sharing reflected improved emotional regulation, protective distancing, or a combination of both.

*“I was very affected early on—my family noticed, and I eventually realized I needed to change.”*

(Nurse 10, 7 years of experience)

*“I used to cry a lot at home. My family helped me see that it wasn’t sustainable.”*

(Nurse 12, 5 years of experience)

### 3.3. The Cost of Empathy

Almost all the nurses shared that prolonged exposure to the suffering of children and their families, particularly during the early stages of their careers, often resulted in emotional exhaustion. Some nurses did not use the term compassion fatigue directly, but they stated that working with these patients caused emotional burden, strain, and exhaustion over time.

#### 3.3.1. Compassion Fatigue

Nurses reported experiencing compassion fatigue and emotional exhaustion, which diminished their capacity for empathy. This was especially prevalent during patient deterioration or death.

*“In the beginning, I was too emotional, and it affected my work.”*

(Nurse 5, 3 years of experience)

*“Compassion fatigue hits hardest when a patient worsens or passes away.”*

(Nurse 2, 14 years of experience)

*“I keep it together at work, but I fall apart emotionally at home.”*

(Nurse 16, 7 years of experience)

#### 3.3.2. Coping Strategies

Over time, nurses developed strategies to cope with the emotional toll of empathic care. These included learning from experienced colleagues, improving communication, and establishing emotional boundaries. Nurses also stated that receiving positive feedback from mothers and children was a significant source of motivation and contributed significantly to their professional satisfaction. Although one participant recalled briefly thinking about quitting, no participant described a current or sustained intention or a concrete plan to leave nursing.

*“I once thought of quitting, but I fell in love with pediatric oncology. It’s hard but spiritually fulfilling.”*

(Nurse 12, 5 years of experience)

*“Compassion is necessary, but it must have limits. We can’t care for others if we’re emotionally depleted.”*

(Nurse 7, 6 years of experience)

## 4. Discussion

This study explored pediatric oncology nurses’ experiences of empathy, revealing that it is a dynamic, multidimensional, and evolving process rather than a fixed personal trait [30]. The findings demonstrate that nurses perceive empathy as essential to meaningful patient and family care yet often struggle to balance emotional engagement with professional boundaries. These results align with prior research indicating that empathy in healthcare is both a cognitive and an emotional skill that requires continuous reflection and regulation [7,26,31].

Consistent with previous studies, nurses commonly defined empathy as “putting oneself in the patient’s shoes” [6,31]. However, during interviews, conceptual confusion emerged, particularly in distinguishing empathy from sympathy. Similar to findings by Sedaghati Kesbakhi and Rohani [32], many participants initially equated empathy with emotional sharing, leading to over-identification and emotional fatigue. This suggests that the emotional component of empathy tends to dominate nursing practice, whereas the cognitive dimension—perspective-taking—remains underdeveloped. Educational interventions that emphasize reflective practice and perspective-taking could therefore help nurses achieve a more balanced and empathic approach [22,23].

Nurses described the constant challenge of maintaining empathy without crossing emotional boundaries. This tension has been widely discussed in the literature [1,4,33]. Participants described strategies such as suppressing emotional expressions or addressing families formally to preserve professional distance. While these coping behaviors may help nurses avoid emotional exhaustion, they can also limit genuine connection and compassion [14]. The findings underscore the need for institutional programs that guide nurses in emotion regulation, boundary-setting, and reflective supervision to sustain empathy while preventing burnout.

Clinical experience emerged as the most influential factor shaping empathy. Early in their careers, nurses often felt overwhelmed by patients’ suffering and uncertain about how to respond emotionally—findings echoed in prior studies [19,33]. Over time, they developed resilience and learned to channel empathy constructively through professional growth. This trajectory supports the notion that empathy is a learnable and developable competence [14,31], and clinical supervision that includes emotional reflection could accelerate this professional maturation process, particularly for novice nurses in high-stress pediatric oncology settings.

Although participants acknowledged communication-related coursework during nursing education, they emphasized that structured empathy training was lacking. Empathy was generally presented superficially under patient-centered care topics, consistent with the findings of Moudatsou et al. [34] and Bas-Sarmiento et al. [23]. Given the emotional complexity of pediatric oncology, undergraduate and in-service programs should incorporate empathy training that combines simulation-based learning, role-play, and reflective writing to strengthen both emotional and cognitive aspects of empathy [22].

An interesting and culturally relevant finding was the influence of family on nurses’ emotional coping. Family feedback appeared to help participants recognize when work-related distress was spilling into home life and to establish clearer boundaries. At the same time, the reported reduction in sharing patient-related stories should not be interpreted uncritically as evidence of growth. In a cultural context such as Türkiye, where family relationships may function as an important source of emotional connection and informal support, sharing less may reflect adaptive emotional regulation and a wish to protect family members, but it may also indicate emotional suppression, withdrawal, or reduced access to support. Similar concerns have been raised in oncology nursing, where self-protective distancing can coexist with unresolved emotional burden [19]. Therefore, the aim should not be to encourage either unrestricted disclosure or silence, but to provide confidential professional spaces—such as reflective supervision, peer debriefing, and staff well-being programs—so that nurses do not have to rely solely on family members to process clinically sensitive and emotionally demanding experiences [24,35,36]. This tension reinforces the importance of culturally responsive emotional support systems that preserve both professional confidentiality and nurses’ meaningful family connections.

Repeated exposure to children’s suffering and death led to compassion fatigue and emotional exhaustion among participants—consistent with previous findings [7,17,26]. One participant recalled considering quitting at one point; however, participants generally described their continued commitment to pediatric oncology nursing and emphasized the meaning, purpose, and spiritual fulfillment they derived from their work. This is consistent with studies linking empathic engagement with job satisfaction and professional commitment [37,38,39]. These accounts indicate that emotional burden and professional commitment can coexist. Because the finding is based on qualitative self-reports, it should not be interpreted as evidence that empathy itself protects nurses from turnover.

Institutional support systems are therefore essential for sustaining empathy while protecting mental health. Programs such as mindfulness-based stress reduction, peer support groups, and reflective supervision can enhance emotional regulation, mitigate compassion fatigue, and reinforce empathic capacity.

## 5. Limitations

This study is a qualitative research study, and the findings are based on the subjective experiences of pediatric oncology nurses and the researchers’ interpretations. The subject’s emotional sensitivity may have led some participants to be cautious when sharing experiences related to empathy, emotional distress, or professional boundaries. The study includes only nurses’ perspectives; the exclusion of patients’ and families’ experiences limits a more comprehensive assessment of the relational dimension of empathy. Furthermore, limiting the sample to specific centers may restrict the generalizability of the findings.

## 6. Conclusions and Clinical Implications

This study contributes to the existing empathy literature by revealing that pediatric oncology nurses experience empathy not merely as a trait one either possesses or lacks, but as an active process negotiated over time through emotional and professional boundaries. The findings show that empathy in pediatric oncology nursing is a complex, multidimensional, and dynamic process. Although nurses viewed empathy as an indispensable element of patient and family care, they described uncertainty about the limits of empathic engagement in professional practice. Clinical experience was described by participants as an important influence on the development and regulation of empathic practice. As they gained experience, nurses described feeling better able to regulate emotional involvement, maintain professional boundaries, and sustain empathic care; however, these accounts represent participants’ self-perceptions and should not be interpreted as objective evidence of improved empathic competence. It is also noteworthy that structured empathy training was reported to be limited during both undergraduate education and in-service professional development. Addressing empathy as a learnable and sustainable professional competency may improve the quality of pediatric oncology care while supporting nurses’ emotional well-being.

Empathy in pediatric oncology nursing is an evolving skill that requires a balance between emotional sensitivity and professional distance. Although nurses view empathy as central to care, conceptual ambiguity, lack of structured education, and emotional strain challenge its practice. Based on these findings, the following recommendations are proposed:

1. Integrate structured empathy training into nursing curricula, addressing both emotional and cognitive dimensions, and emphasizing professional boundary maintenance.
2. Develop mentorship and reflective supervision programs to help novice nurses manage emotional demands and cultivate professional empathy.
3. Establish institutional mechanisms—peer support, debriefing sessions, and mindfulness training—to sustain empathy while preventing compassion fatigue.
4. Acknowledge cultural factors in emotional coping and resilience, recognizing both the supportive role and potential limits of family-based sharing, while ensuring nurses have access to confidential professional support systems.

## Figures and Tables

**Table 1:** Demographic characteristics of participants.

Characteristics (n = 17)	Frequency	%
Gender		
Female	17	100.0
Educational level		
Bachelor’s	15	88.2
Master’s	2	11.8
Work experience on pediatric oncology unit ^a^		
2–5 years	7	41.2
6–10 years	6	35.3
More than 9 years	4	23.4
Types of hospitals		
Public hospital	3	17.7
University hospital	3	17.7
Private hospital	11	64.3
Age ^a,b^	28.89 ± 3.91 (24–36)	

^a^ Years. ^b^ Mean ± standard deviation, range.

## Data Availability

The data that support the findings of this study are available on request from the corresponding author. The data are not publicly available due to privacy or ethical restrictions.
